# Association between Estimated Small Dense Low-Density Lipoprotein Cholesterol and Occurrence of New Lesions after Percutaneous Coronary Intervention in Japanese Patients with Stable Angina and Receiving Statin Therapy

**DOI:** 10.31083/j.rcm2506218

**Published:** 2024-06-17

**Authors:** Daisuke Kanda, Akihiro Tokushige, Mitsuru Ohishi

**Affiliations:** ^1^Department of Cardiovascular Medicine and Hypertension, Graduate School of Medical and Dental Sciences, Kagoshima University, 890‑8520 Kagoshima, Japan

**Keywords:** stable angina, estimated small dense LDL-C, residual cardiovascular risk, percutaneous coronary intervention, statins, diabetes mellitus

## Abstract

**Background:**

Low-density lipoprotein cholesterol (LDL-C) is considered the most important risk factor for coronary artery disease (CAD). Although lipid-lowering therapy using high-intensity statins for patients with stable CAD is one of the cornerstones of medication therapy, there is still a risk of residual cardiovascular events, even after controlling for LDL-C. Recently, attention has focused on the association between small dense LDL-C as a residual risk factor for CAD, and it has been reported that a formula can be used to calculate the small LDL-C.

**Methods:**

We investigated the association between estimated small dense LDL-C (Esd LDL-C) and the occurrence of new lesions with myocardial ischemia ≤2 years after percutaneous coronary intervention (PCI) in 537 patients with stable angina who underwent PCI. In this study, all patients had been prescribed statins. This study was based on previously reported data regarding the relationship between non-high-density lipoprotein cholesterol levels and stable angina pectoris after PCI.

**Results:**

Revascularization, including new lesions and in-stent restenosis, and new lesions appeared in 130 and 90 patients, respectively, ≤2 years after PCI. Age, diabetes mellitus (DM), LDL-C, and Esd LDL-C were associated with the occurrence of revascularization and new lesions ≤2 years after PCI. Multivariate logistic regression analysis models revealed that Esd LDL-C [odds ratio (OR) 1.03, 95% confidence interval (CI) 1.004–1.048, *p* = 0.020; and OR 1.03, 95% CI 1.009–1.057, *p* = 0.007, respectively] were associated with the revascularization and occurrence of new lesions ≤2 years after PCI.

**Conclusions:**

As well as total cholesterol and LDL-C, Esd LDL-C was an independent risk factor for the revascularization and occurrence of new lesions ≤2 years after PCI for stable angina in Japanese patients receiving statin therapy. In patients with stable angina who are on lipid-lowering therapy with statins, calculating the Esd LDL-C may provide useful information for predicting revascularization and the occurrence of new lesions.

## 1. Introduction

Ischemic heart disease, represented by coronary artery disease (CAD), is a major cause of death due to cardiovascular diseases [1], and hypercholesterolemia is one of the most important causal factors for atherosclerotic cardiovascular disease observed in CAD.

Percutaneous coronary intervention (PCI) using second-generation drug-eluting stents (2nd DES) is one of the major procedures for coronary revascularization in patients with CAD [2]. Despite clinical advances in PCI and medical therapies, controlling cardiovascular events remains a major problem.

Low-density lipoprotein cholesterol (LDL-C) is considered the major risk factor for cardiovascular disease and remains the primary target of current cardiovascular risk reduction strategies [3]. Similar to the results of many epidemiological studies in Europe and the United States, the cohort studies for Japanese patients confirmed that the hazard ratio for CAD incidence and death increases as LDL-C levels increase [4].

Lipid-lowering therapy using high-intensity statins for patients with stable CAD is the cornerstone of optimal medical therapy (OMT). A meta-analysis using 170,000 participants in 26 previous randomized controlled trials (RCTs) revealed the effect of intensive lipid-lowering therapy regardless of the baseline LDL-C level [5].

Therefore, lipid-lowering therapy is an essential strategy for preventing CAD [3, 6]. In Japan, an LDL-C target of <70 mg/dL is recommended for cardiovascular high-risk patients [7]. However, a residual risk of cardiovascular events persists even after achieving this target.

Conversely, some argue that non-high-density lipoprotein cholesterol (non-HDL-C), which is easily calculated without an estimate for measurement, is a more useful predictor of atherosclerotic disease than LDL-C since it is the non-HDL-C that contains the atherosclerosis-inducing lipoproteins [8]. Several epidemiological studies have reported an association between non-HDL-C and CAD. We also reported that non-HDL-C is a useful parameter for predicting the development of new lesions in patients with stable angina who underwent PCI [9].

Small dense LDL-C (sd LDL-C), a component of non-HDL-C, tends to accumulate in the vascular wall and oxidizes to LDL-C, which is considered highly atherosclerosis-inducing [10, 11]. Recently, a simple method for measuring sd LDL-C using LDL-C and triglyceride (TG) levels has been reported, and this method does not require the direct measurement of sd LDL-C [12]. However, the relationship between sd LDL-C calculated using this method and the occurrence of new lesions in patients with stable angina undergoing PCI is unknown.

Thus, in this study, we aimed to investigate the clinical significance of LDL-C and calculate the estimated small dense LDL-C (Esd LDL-C) using a formula for the occurrence of revascularization and new lesions in patients with stable angina who were prescribed statins and underwent PCI.

## 2. Materials and Methods

### 2.1 Study Population 

This retrospective single-center cohort study at Kagoshima University Hospital included 621 patients with stable angina who had been treated with intensive statins for at least 2 weeks after admission and underwent successful elective PCI with a 2nd DES between January 2010 and December 2018. Intravascular ultrasound or optical coherence tomography were used in all cases as imaging modalities during PCI. All patients were administered statins before the PCI, regardless of dyslipidemia. The exclusion criteria included patients with acute coronary syndrome, those undergoing hemodialysis, those with insufficient blood test results, and those with fasting serum TG levels >400 mg/dL. Finally, 23 patients were excluded using the above criteria. We also excluded 61 patients who were lost to follow-up after discharge. Baseline demographic data, including cardiovascular risk factors, such as current smoking and diabetes mellitus (DM), were registered.

This study was approved by the Research and Ethics Committee of Kagoshima University Hospital (approval number 200144疫). It was conducted in accordance with the ethical principles of the 1975 Declaration of Helsinki. All patients provided written informed consent before enrolment.

### 2.2 Measurements

Laboratory values were obtained upon admission and before PCI. Blood samples of the serum concentrations of high-sensitivity C-reactive protein, total cholesterol (TC), HDL-C, TG, and creatinine were collected after a 12-hour fast. The estimated glomerular filtration rate (eGFR) in this study was calculated using the Modification of Diet in Renal Disease equation with coefficients modified for Japanese patients as follows:

$$\begin{array}{c} \mathrm{eGFR}\,\left(\mathrm{mL}/\min/1.73\,m^{2}\right)=194\times \mathrm{serum}\,\mathrm{Cr}\,\left(\mathrm{mg}/\mathrm{dL}\right)\\ -1.094\times \mathrm{age}\left(\mathrm{years}\right)-0.287\left(\times 0.739\mathrm{for}\mathrm{female}\mathrm{subjects}\right) \end{array}$$

[13].

Estimated LDL-C, large buoyant LDL-C (lbLDL-C), and estimated sd LDL-C were calculated by using Sampson’s formulas [12]:

$$\begin{array}{c} \text{ Estimated }\,\mathrm{LDL}-C\,\left(\mathrm{ELDL}-C\right)=\mathrm{TC}/0.948-\mathrm{HDL}-\\ C/0.971-(\mathrm{TG}/8.56+\left[\mathrm{TG}\times \mathrm{non}-\mathrm{HDL}-C\right]/2140\\ -\mathrm{TG}\times \mathrm{TG}/16100)-9.44 \end{array}$$

(2) $$\begin{array}{c} \text{ Large buoyant }\left(\mathrm{lb}\right)\mathrm{LDL}-C=1.43\times \mathrm{ELDL}-C-[0.14\\ \times \left(\log\mathrm{of}\mathrm{TG}\right)\times \mathrm{ELDL}-C]-8.99 \end{array}$$

(3) $$\begin{array}{c} \text{ Estimated sd }\,\mathrm{LDL}-C\,\left(\mathrm{EsdLDL}-C\right)\\ =\mathrm{ELDL}-C-\mathrm{lbLDL}-C \end{array}$$

### 2.3 Definitions

Current smokers were defined as those who smoked actively at the time of admission. In this study, the statin treatments were prescribed according to the PATROL (Pitavastatin, Atorvastatin, and Rosuvastatin for Safety and Efficacy (Quantity and Quality of LDL)) trial protocol, which was defined as atorvastatin ≥10 mg/day, rosuvastatin ≥2.5 mg/day, and pitavastatin ≥2 mg/day [14].

The guidelines of the Japan Atherosclerosis Society recommend that LDL-C <70 mg/dL is the target of secondary prevention with high-risk factors for CAD recurrence for familial hypercholesterolemia, acute coronary syndrome, or DM [7].

Follow-up coronary angiography was conducted after 9 months. New lesions were defined as having >75% stenosis on angiography and myocardial ischemia. Myocardial ischemia was estimated using the fractional flow reserve or myocardial perfusion single-photon emission computed tomography. Revascularization was defined as PCI for the new lesions and in-stent restenosis (ISR). The presence of new lesions after PCI was defined the incidences of clinically-driven revascularization for the non-target lesion.

All patients received and continued to be treated with statins during follow-ups, which occurred at our hospital or by the patient’s physicians.

### 2.4 Statistical Analysis 

For descriptive statistics, frequencies (percentages) were used for categorical variables, and mean ± standard deviation, or median and interquartile range (IQR) were used for continuous variables. The incidence rates for categorical variables expressed as percentages were compared using Fisher’s exact test. Continuous variables between the revascularization (+) and revascularization (-) groups were compared, and between the new lesion (+) and new lesion (-) groups using Student’s *t*-test (normal distribution) or Wilcoxon rank-sum test (non-normal distribution). We analyzed the relationship between the revascularization (+) and new lesion (+) groups and background factors using logistic regression analysis with the inclusion of odds ratios (ORs) and 95% confidence intervals (CIs). In addition, multivariate logistic regression modeling with relevant factors analyzed independent associations between the revascularization (+) and new lesion (+) groups and baseline characteristics. The multivariate analysis model included variables with statistical significance in the univariate analysis. We added confounding variables to our model for each of the multiple pairs and compared the adjusted odds ratios to evaluate the robustness of our results and assess the impact of confounding variables. Receiver-operating characteristic (ROC) curve analyses to evaluate the TC, LDL-C, and Esd LDL-C for revascularization and occurrence of new lesions ≤2 years after PCI were performed, and compared these areas under the curves (AUCs). Statistical significance was set at *p* < 0.05. Statistical analyses were performed using the SAS software (JMP version 17.0, SAS Institute Inc., Cary, NC, USA).

## 3. Results

### 3.1 Baseline Characteristics and Comparison of Baseline Characteristics of Study Patients According to Revascularization (New Lesions + ISR) and Occurrence of New Lesions ≤2 Years after PCI 

The clinical characteristics of patients at baseline and a comparison between the baseline characteristics of study patients according to revascularization (new lesions + ISR) and occurrence of new lesions ≤2 years after PCI are shown in Table 1. A total of 130 patients (24%) required revascularization due to the occurrence of new lesions and/or in-stent restenosis after PCI. Of the 537 patients recruited, 90 patients (17%) showed new lesions ≤2 years after PCI.

**Table 1.** **Comparison of baseline characteristics of study patients according to revascularization (new lesions + ISR) and occurrence of new lesions ≤2 years after PCI**.

			Revascularization (+) (new lesions + ISR) ≤2 years			New lesion (+) ≤2 years		
Variables		Overall	Revascularization (+) group	Revascularization (-) group	*p value*	New lesion (+) group	New lesion (-) group	*p value*
		(n = 537)	(n = 130)	(n = 407)		(n = 90)	(n = 447)	
Age, years		69 [64–76]	67 [60–75]	70 [64–77]	0.007	66 [59–75]	70 [64–77]	0.006
Sex: male, n (%)		392 (73)	97 (75)	295 (72)	0.73	67 (74)	325 (73)	0.73
Body mass index, kg/$m^{2}$		23.8 [21.5–6.2]	24.3 [21.8–26.2]	23.9 [21.3–26.1]	0.26	23.8 [21.6–26.3]	23.9 [21.5–26.1]	0.52
Risk factors, n (%)								
	Hypertension	438 (82)	108 (83)	330 (81)	0.70	73 (81)	365 (82)	0.90
	Diabetes mellitus	313 (58)	91 (70)	222 (55)	0.002	62 (69)	251 (56)	0.025
	Dyslipidemia	408 (76)	99 (76)	309 (76)	1.00	68 (76)	340 (76)	0.92
	Current smoker	63 (12)	18 (14)	45 (11)	0.43	13 (15)	50 (11)	0.36
	Smoking history	81 (15)	28 (22)	53 (13)	0.024	18 (20)	63 (14)	0.20
Medication, n (%)								
	Oral anti-coagulation	65 (12)	19 (15)	46 (11)	0.35	13 (14)	52 (12)	0.46
	Calcium-channel blocker	229 (43)	60 (46)	169 (42)	0.36	42 (47)	187 (42)	0.40
	ACEI	101 (19)	25 (19)	76 (19)	0.89	14 (16)	87 (19)	0.39
	ARB	225 (42)	56 (43)	169 (42)	0.76	39 (43)	186 (42)	0.76
	β-blocker	185 (34)	47 (36)	138 (34)	0.67	31 (34)	154 (34)	0.99
	Ezetimibe	40 (7)	9 (7)	31 (8)	0.85	6 (7)	34 (7)	0.99
Laboratory data								
	hs-CRP, mg/L	1.2 [0.4–3.2]	1.4 [0.4–3.1]	1.1 [0.4–3.2]	0.91	1.7 [0.6–3.7]	1.1 [0.4–3.1]	0.159
	TC, mg/dL	155 [136–81]	163 [141–193]	153 [135–175]	0.002	168 [140–197]	153 [135–176]	0.001
	LDL-C, mg/dL	83 [66–105]	95 [70–118]	81 [66–101]	0.001	97 [71–124]	81 [66–101]	<0.001
	Esd LDL-C, mg/dL	26 [22–33]	29 [23–37]	26 [22–32]	0.002	29 [24–39]	26 [22–32]	<0.001
	HDL-C, mg/dL	47 [40–58]	48 [39–58]	47 [40–59]	0.086	47 [38–55]	48 [40–59]	0.21
	TG, mg/dL	109 [82–154]	114 [90–168]	106 [80–151]	0.075	114 [92–181]	108 [80–151]	0.066
	Remnant (TC-HDL-C-LDL-C)	19 [14–25]	19 [14–26]	19 [14–24]	0.49	19 [15–27]	18 [14–25]	0.157
	Uric acid, mg/dL	5.9 [4.9–6.8]	6.2 [5.2–6.8]	5.8 [4.9–6.8]	0.19	6.1 [4.8–6.7]	5.9 [5.0–6.8]	0.99
	FPG, mg/dL	110 [96–133]	115 [99–139]	109 [94–131]	0.054	116 [95–135]	109 [96–133]	0.31
	HbA1c, %	6.3 [5.8-7.0]	6.4 [5.9–7.0]	6.2 [5.8–7.0]	0.058	6.4 [5.9–7.4]	6.2 [5.8–6.9]	0.079
	eGFR, mL/min/1.73 $m^{2}$	61.4 [47.5–75.2]	63.8 [49.7–77.9]	60.1 [47.0–73.8]	0.105	64.9 [50.9–77.8]	59.7 [47.2–74.1]	0.065
LVEF, %		58.3 [48.7–66.2]	59 [52–66]	58 [47–66]	0.28	57.7 [50.8–66.2]	58.3 [47.9–66.2]	0.76

Values are presented as numbers, percentages, or medians with interquartile ranges. Abbreviations: ISR, in-stent restenosis; ACEI, angiotensin-converting enzyme inhibitor; ARB, angiotensin II receptor blocker; Esd LDL-C, estimated small dense low-density lipoprotein cholesterol; eGFR, estimated glomerular filtration rate; FPG, fasting plasma glucose; HbA1c, glycated hemoglobin; HDL-C, high-density lipoprotein cholesterol; hs-CRP, high-sensitivity C-reactive protein; LVEF, left ventricular ejection fraction; LDL-C, low-density lipoprotein cholesterol; PCI, percutaneous coronary intervention; TC, total cholesterol; TG, triglyceride.

Age was lower in the revascularization (+) and new lesion (+) ≤2 years groups than in the revascularization (-) group and new lesion (-) ≤2 years groups. Smoking history was significantly higher in only the revascularization (+) group than in the revascularization (-) group (22% vs. 13%; *p* = 0.024).

The frequencies of DM, TC, LDL-C, and Esd LDL-C were significantly higher in the revascularization (+) group compared to the revascularization (-) group (DM, *p* = 0.002; TC, *p* = 0.002; LDL-C, *p* = 0.001; Esd LDL-C, *p* = 0.002).

In the new lesion (+) ≤2 years group, the frequency of DM was significantly higher compared with the new lesion (-) ≤2 years group (69% vs. 56%; *p* = 0.025). Furthermore, the level of TC, LDL-C, and Esd LDL-C were significantly higher than those in the new lesion (-) ≤2 years group (TC, *p* = 0.001; LDL-C, *p* ≤ 0.001; Esd LDL-C, *p* ≤ 0.001, respectively).

### 3.2 Effect of Baseline Characteristics on Revascularization, Including New Lesions and In-Stent Restenosis

We performed univariate logistic regression analysis to investigate the impact of background factors on revascularization and occurrence of new lesions ≤2 years after PCI (**Supplementary Table 1**). Age, DM, smoking history, TC, LDL-C, and Esd LDL-C were associated with the revascularization group.

Furthermore, age, DM, TC, LDL-C, Esd LDL-C, and HbA1c were associated with the new lesion (+) ≤2 years group (**Supplementary Table 1**).

A multivariate logistic regression analysis was performed (Tables 2,3). LDL-C and Esd LDL-C were components of TC, and it was not appropriate to include them in the multivariate logistic analysis simultaneously, so we chose three models.

**Table 2.** **Multivariate logistic analysis models to predict the occurrence of revascularization (new lesions + ISR) after PCI as determined by multivariate logistic analysis**.

	OR	Model 1		OR	Model 2		OR	Model 3	
		95% CI	*p value*		95% CI	*p value*		95% CI	*p value*
Age, years	0.99	0.97–1.01	0.20	0.99	0.97–1.01	0.27	0.99	0.97–1.01	0.28
Sex: male	0.95	0.59–1.54	0.21	0.95	0.58–1.56	0.85	0.93	0.57–1.50	0.76
Diabetes mellitus	2.05	1.32–3.16	0.001	2.15	1.38–3.34	<0.001	1.97	1.28–3.04	0.021
Smoking history	1.97	1.15–3.39	0.014	1.99	1.15–3.42	0.013	2.04	1.18–3.50	0.010
TC	1.007	1.001–1.013	0.016	-	-	-	-	-	-
LDL-C	-	-	-	1.009	1.003–1.017	0.007	-	-	-
Esd LDL-C	-	-	-	-	-	-	1.026	1.004–1.048	0.020

ISR, in-stent restenosis; PCI, percutaneous coronary intervention; OR, odds ratio; TC, total cholesterol; LDL-C, low-density lipoprotein cholesterol; Esd LDL-C, estimated small dense low-density lipoprotein cholesterol. Model 1, adjusted for age, sex (male), diabetes mellitus, smoking history, and TC. Model 2, adjusted for age, sex (male), diabetes mellitus, smoking history, and LDL-C. Model 3, adjusted for age, sex (male), diabetes mellitus, smoking history, and Esd LDL-C.

**Table 3.** **Multivariate logistic analysis models to predict the occurrence of new lesions ≤2 years after PCI**.

	OR	Model 1		OR	Model 2		OR	Model 3	
		95% CI	*p value*		95% CI	*p value*		95% CI	*p value*
Age, years	0.98	0.96–1.004	0.124	0.99	0.96–1.01	0.236	0.99	0.96–1.01	0.22
Sex: male	1.08	0.63–1.84	0.772	1.05	0.61–1.81	0.85	1.06	0.62–1.81	0.82
Diabetes mellitus	1.71	1.05–2.78	0.032	1.71	1.04–2.82	0.034	1.62	0.99–2.65	0.048
TC	1.008	1.002–1.015	0.014	-	-	-	-	-	-
LDL-C	-	-	-	1.013	1.005–1.021	<0.001	-	-	-
Esd LDL-C	-	-	-	-	-	-	1.033	1.009–1.057	0.007

PCI, percutaneous coronary intervention; OR, odds ratio; TC, total cholesterol; LDL-C, low-density lipoprotein cholesterol; Esd LDL-C, estimated small dense low-density lipoprotein cholesterol. Model 1, adjusted for age, sex (male), diabetes mellitus, and TC. Model 2, adjusted for age, sex (male), diabetes mellitus, and LDL-C. Model 3, adjusted for age, sex (male), diabetes mellitus, and Esd LDL-C.

The presence of DM and a smoking history were independent risk factors for revascularization. TC, LDL-C, and Esd LDL-C were significantly associated with revascularization (TC, *p* = 0.016; LDL-C, *p* = 0.007; Esd LDL-C, *p* = 0.020) (Table 2). In Model 1 and Model 2, DM was the independent risk factor for the occurrence of new lesions (DM: Model 1, odds ratio (OR) 1.71, 95% CI 1.05–2.78, *p* = 0.032; Model 2, OR 1.71, 95% CI 1.04–2.82, *p* = 0.034). Additionally, in Model 3, only Esd LDL-C was an independent risk factor for the occurrence of new lesions ≤2 years after PCI (*p* = 0.007), meaning DM was not (Model 3, OR 1.62, 95% CI 0.99–2.65, *p* = 0.048) (Table 3).

### 3.3 Predictive Values of Esd LDL-C for Revascularization and the Occurrence of New Lesions ≤2 Years after PCI 

**Fig. 1.**
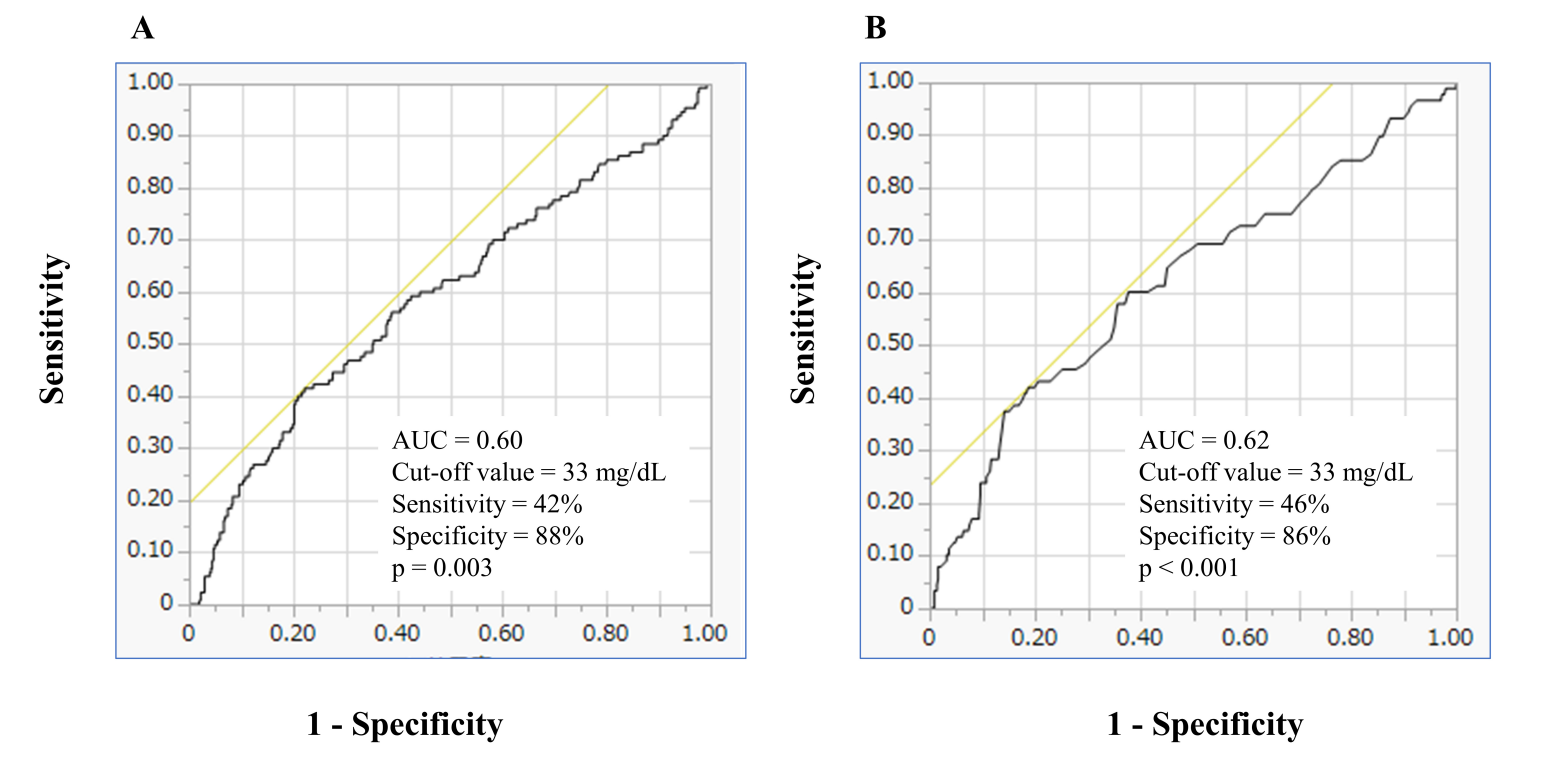
**Receiver-operating characteristics curves of Esd LDL-C for predicting revascularization (A) and the occurrence of new lesions ≤2 years after PCI (B).** AUC, area under the curve; Esd LDL-C, estimated small dense low-density lipoprotein cholesterol; PCI, percutaneous coronary intervention.

**Fig. 2.**
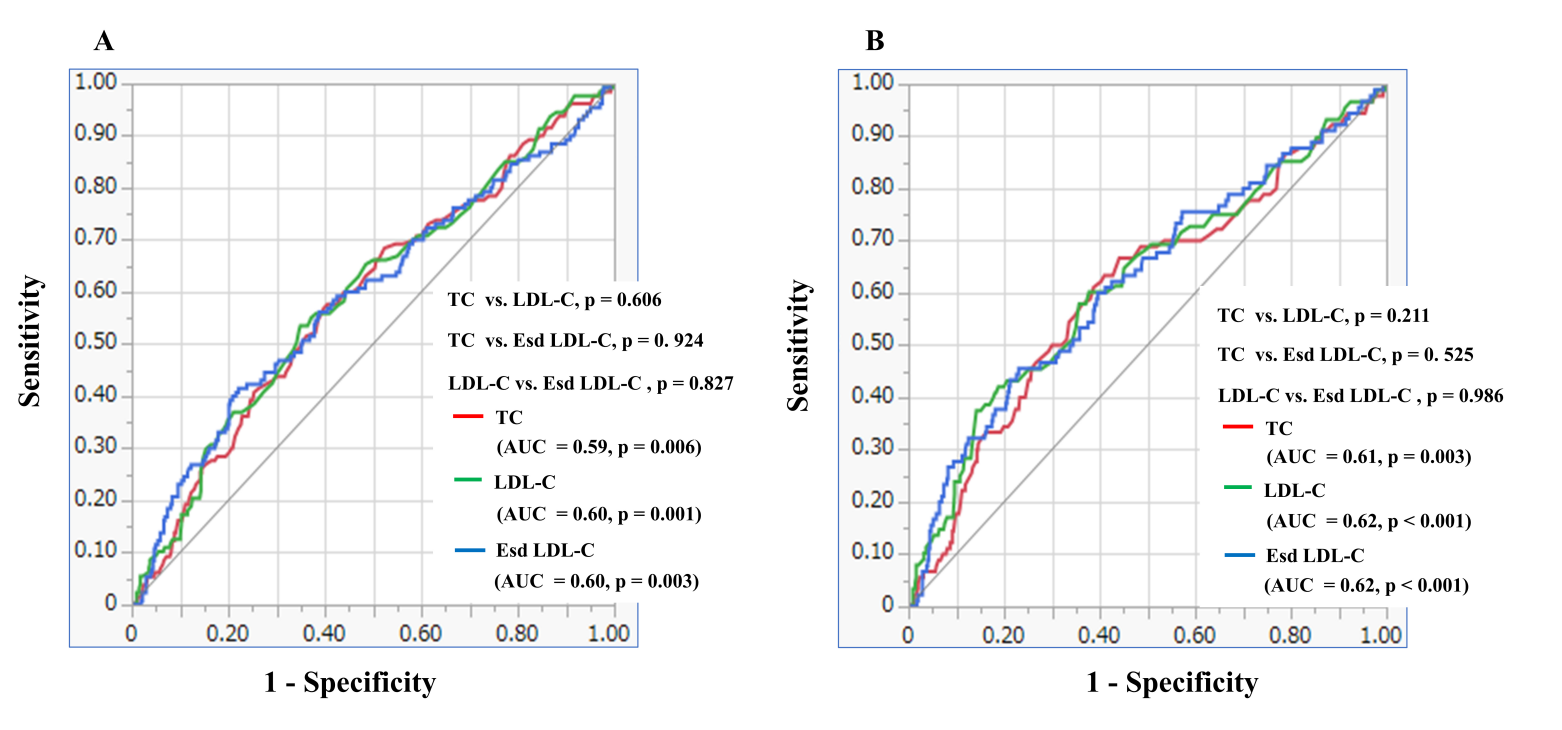
**Receiver-operating characteristics curves for predicting revascularization (A) and the occurrence of new lesions ≤2 years after PCI (B).** AUC, area under the curve; LDL-C, low-density lipoprotein cholesterol; Esd LDL-C, estimated small dense LDL-C; PCI, percutaneous coronary intervention; TC, total cholesterol.

These results showed that Esd LDL-C offers a potent independent risk factor of revascularization and the occurrence of new lesions ≤2 years after PCI. We, therefore, analyzed ROC curves to evaluate the effect of Esd LDL-C for predicting revascularization and the occurrence of new lesions ≤2 years after PCI. ROC cutoffs for Esd LDL-C in revascularization and the occurrence of new lesions were both 33 (AUC = 0.60, *p* = 0.003 and AUC = 0.62, *p* < 0.001, respectively) (Fig. 1). Furthermore, comparisons of ROC curves for TC, LDL-C and Esd LDL-C for predicting revascularization and the occurrence of new lesions ≤2 years after PCI showed no significant difference in AUCs between TC, LDL-C and Esd LDL-C (Fig. 2).

## 4. Discussion

This study demonstrated that Esd LDL-C was an independent risk factor for the revascularization and occurrence of new lesions ≤2 years after PCI in Japanese patients with stable angina treated with statins.

Given the Clinical Outcomes Utilizing Revascularization and Aggressive Drug Evaluation (COURAGE) and International Study of Comparative Health Effectiveness With Medical and Invasive Approaches (ISCHEMIA) trial results, OMT is considered the cornerstone of CAD management [15, 16].

Thus, the current baseline treatment concept for patients with stable CAD is to reduce LDL-C levels using high-intensity statins, which is recommended [3, 6, 7].

However, even when LDL-C levels are well-controlled, a residual risk of cardiovascular events exists. Unfortunately, although PCI is suitable for the revascularization of target lesions, it does not have the effect of suppressing new lesions. Therefore, suppressing the development of new lesions after PCI is essential for the prognosis of CAD patients.

DM is a well-known risk factor for CAD. It has been reported that coronary artery plaques are more likely to progress in patients with DM, and plaque regression rates are significantly lower despite controlled LDL-C levels [17, 18]. Furthermore, DM patients are more likely to exhibit lipid abnormalities, such as hypercholesterolemia, hypertriglyceridemia, low HDL-C levels, and small, dense LDL particles, and often have comorbidities of dyslipidemia [19].

Additionally, DM has been reportedly associated with in-stent restenosis. The pathogenesis of in-stent restenosis in patients with DM involves vascular injury followed by repair processes, including endothelialization, inflammation, neointimal hyperplasia, and other factors [20]. Therefore, even after PCI, patients with DM require constant attention for the occurrence of restenosis and new lesions, which is a clinically important issue.

Lipid-lowering therapy has been identified as vital for preventing CAD [3, 6, 7].

Indeed, sd LDL is small and highly dense among LDL particles and has a high potential to facilitate atherogenic processes. It is considered that sd LDL has high susceptibility to oxidation, high endothelial permeability, and decreased hepatic LDL receptor affinity [11, 21].

In patients with metabolic syndrome, represented by DM and dyslipidemia, the measured LDL-C may not accurately reflect LDL particle concentrations and their effects on CAD due to the discordance of sd LDLs [22]. Furthermore, dyslipidemia in patients with DM often has high serum TG concentrations, sd LDL-C particles, and low HDL-C concentrations.

We previously reported that non-HDL-C is more useful than LDL-C in predicting the presence of new lesions in patients with stable angina pectoris [9]. This is because we considered that (1) non-HDL-C can be simply and accurately calculated regardless of fasting, and (2) non-HDL-C represents all atherogenic lipoproteins.

TG-rich LDLs lead to the production of sd LDLs with small particles and high specific gravity. Furthermore, their residence time in the blood is reported to be approximately 1.5 times longer than normal LDL-C [23].

The causes of sd LDL-C formation are complex and can be influenced by a combination of genetic, metabolic, and lifestyle factors [24]. Thus, identifying and addressing these factors is essential for managing the risk of cardiovascular diseases associated with sd LDL particles.

The sd LDL concentration is measured using either ultracentrifugation or gradient gel electrophoresis. However, these methods are not usually routine diagnostic tests because of the intensive labor involved. Our findings revealed that Esd LDL-C was a useful factor, as was LDL-C in the revascularization and occurrence of new lesions after PCI in patients with stable angina pectoris.

Previous studies failed to prove the clinical efficacy of lipid-lowering therapy based on Western target LDL-C levels of <55 mg/dL in East Asian AMI patients. In a study of Korean AMI patients, a target LDL-C level of <55 mg/dL had no additional benefits than a target LDL-C level of <70 mg/dL. Our study targeted Japanese patients with CAD. We believe that in addition to LDL-C lowering therapy, calculating Esd LDL-C and subjecting it to risk assessment may be an option for finding treatments that can suppress the occurrence of new lesions [25].

In this study, Esd LDL-C may be useful in predicting the residual risk after PCI in patients with stable angina treated with statins. There are limits to the lipid-lowering therapy that can be improved with statins [26]. In addition, it has been reported that statins do not reduce sd LDL-C as much as total LDL-C [27]. In high-risk patients receiving statins who have had a coronary event, there is room to measure Esd LDL-C and consider more robust lipid-lowering therapies, such as the additional use of PSCK9 inhibitors, which have the effect of reducing sd LDL-C [28, 29]. Therefore, establishing treatment goals for patients with stable angina based on Esd LDL-C scores may be helpful. These findings should be evaluated in future prospective studies.

### Limitations

This study has several limitations. First, this retrospective, single-center study involved a relatively small cohort of patients. Second, the dose and specific types of statins were not standardized. Third, this current study only included patients with stable angina; thus, future research on patients with acute coronary syndrome is required. Fourth, the Esd LDL-C calculation formula used in this study needs to be prospectively verified in a large-scale cohort in the future, as there are no data on Japanese individuals. Fifth, despite the existence of guidelines for CAD patients, lipid-lowering therapy robust enough to achieve the target value range was limited in this study. Therefore, in the future, we would like to perform further lipid enrichment therapy and verify whether this data can be further utilized.

## 5. Conclusions

As well as TC and LDL-C, Esd LDL-C was an independent risk factor for the revascularization and occurrence of new lesions ≤2 years after PCI for stable angina in Japanese patients receiving statin therapy. In lipid-lowering therapy for CAD patients with LDL-C as the main target, measuring Esd LDL-C may provide useful information for predicting revascularization and the occurrence of new lesions. In the future, it is necessary to verify further whether these data can be used in a large population managed with sufficiently strong lipid-lowering therapy based on current guidelines.

## Data Availability

The datasets used and analyzed in this study are available from the corresponding author upon reasonable request.

## Abbreviations

DM, diabetes mellitus; LDL-C, low-density lipoprotein cholesterol; Esd LDL-C, estimated small dense low-density lipoprotein cholesterol; PCI, percutaneous coronary intervention; sd LDL-C, small dense low-density lipoprotein cholesterol; TC, total cholesterol; TG, triglycerides.

## Supplementary Material

Supplementary material associated with this article can be found, in the online version, at https://doi.org/10.31083/j.rcm2506218.
