# Supplementary material for: Association between Estimated Small Dense Low-Density Lipoprotein Cholesterol and Occurrence of New Lesions after Percutaneous Coronary Intervention in Japanese Patients with Stable Angina and Receiving Statin Therapy
Source: Rev Cardiovasc Med. 2024 Jun 17;25(6):218. doi: 10.31083/j.rcm2506218 (PMC11270076; doi:10.31083/j.rcm2506218)
Supplement: Supplementary file 1 [file 2153-8174-25-6-218-s1.docx]

**Supplementary Table 1**

**Univariate logistic analysis for the occurrence of revascularization (new lesions + ISR) and new lesion ≤ 2 years after PCI**

|  | Revascularization (new lesions + ISR) N (%) =130 (8) | | | New lesion (+) **≤** 2 years  N (%) = 90 (17) | | |
| --- | --- | --- | --- | --- | --- | --- |
|  | OR | 95%CI | *p value* | OR | 95%CI | *p value* |
| Age, years | 0.98 | 0.95–0.99 | 0.024 | 0.97 | 0.95–0.99 | 0.020 |
| Sex: male, n (%) | 1.12 | 0.71–1.75 | 0.63 | 1.09 | 0.66–1.86 | 0.73 |
| Body mass index, kg/m^2^ | 1.04 | 0.99–1.09 | 0.170 | 1.04 | 0.98–1.10 | 0.20 |
| Risk factors, n (%) |  |  |  |  |  |  |
| Hypertension | 1.15 | 0.68–1.92 | 0.61 | 0.96 | 0.55–1.77 | 0.90 |
| Diabetes mellitus | 1.94 | 1.27–2.97 | 0.002 | 1.73 | 1.08–2.84 | 0.027 |
| Dyslipidemia | 1.01 | 0.64–1.61 | 0.96 | 1.02 | 0.58–1.71 | 0.92 |
| Current smoker | 1.30 | 0.72–2.35 | 0.37 | 1.36 | 0.68–2.55 | 0.36 |
| History of smoking | 1.83 | 1.09–3.04 | 0.020 | 1.52 | 0.83–2.67 | 0.16 |
| Medication, n (%) |  |  |  |  |  |  |
| Oral anticoagulation | 1.34 | 0.75–2.39 | 0.31 | 1.28 | 0.64–2.40 | 0.45 |
| Calcium-channel blocker | 1.21 | 0.88–1.79 | 0.35 | 1.22 | 0.77–1.92 | 0.40 |
| ACEI | 1.04 | 0.62–1.71 | 0.89 | 0.76 | 0.40–1.37 | 0.39 |
| ARB | 1.07 | 0.71–1.59 | 0.75 | 1.07 | 0.68–1.69 | 0.76 |
| β-blocker | 1.10 | 0.73–1.66 | 0.64 | 1.00 | 0.61–1.60 | 0.99 |
| Ezetimibe | 0.89 | 0.41–1.94 | 0.78 | 0.86 | 0.32–1.98 | 0.75 |
| Laboratory data |  |  |  |  |  |  |
| hs-CRP, mg/L | 0.99 | 0.98–1.01 | 0.25 | 0.99 | 0.98–1.01 | 0.51 |
| TC, mg/dL | 1.01 | 0.99–1.02 | 0.054 | 1.01 | 1.00–1.02 | 0.003 |
| LDL-C, mg/dL | 1.01 | 1.004–1.017 | 0.001 | 1.015 | 1.017–1.02 | 0.021 |
| Esd LDL-C, mg/dL | 1.03 | 1.01–1.05 | 0.003 | 1.04 | 1.02–1.06 | < 0.001 |
| HDL-C, mg/dL | 0.99 | 0.98–1.01 | 0.91 | 0.99 | 0.97–1.00 | 0.149 |
| TG, mg/dL | 1.002 | 0.999–1.005 | 0.128 | 1.003 | 0.999–1.007 | 0.065 |
| Remnant (TC-HDL-C-LDL-C) | 1.00 | 0.97–1.12 | 0.96 | 1.00 | 0.99–1.02 | 0.57 |
| Uric acid, mg/dL | 1.06 | 0.93–1.21 | 0.40 | 0.99 | 0.84–1.16 | 0.89 |
| FPG, mg/dL | 1.00 | 0.99–1.01 | 0.126 | 1.00 | 0.99–1.00 | 0.26 |
| HbA1c, % | 1.18 | 0.99–1.39 | 0.054 | 1.23 | 1.02–1.47 | 0.028 |
| eGFR, mL/min/1.73 m^2^ | 1.01 | 0.99–1.02 | 0.186 | 1.01 | 0.99–1.02 | 0.102 |
| LVEF, % | 1.01 | 0.99–1.03 | 0.154 | 1.01 | 0.98–1.02 | 0.56 |

Values are presented as numbers, percentages, or medians with interquartile ranges.

Abbreviations: ACEI, angiotensin-converting enzyme inhibitor; ARB, angiotensin II receptor blocker; CI, confidence interval; Esd LDL-C, estimated small dense low-density lipoprotein cholesterol; eGFR, estimated glomerular filtration rate; FPG, fasting plasma glucose; HbA1c, glycated hemoglobin; HDL-C, high-density lipoprotein cholesterol; hs-CRP, high-sensitivity C-reactive protein; LVEF, left ventricular ejection fraction; LDL-C, low-density lipoprotein cholesterol; OR, odds ratio; PCI, percutaneous coronary intervention; TC, Total cholesterol; TG, Triglyceride
